# How Long Can You Delay? Curve Progression While Awaiting Vertebral Body Tethering Surgery

**DOI:** 10.3390/jcm13082209

**Published:** 2024-04-11

**Authors:** Christina Regan, M. Bryant Transtrum, Bharadwaj Jilakara, Todd A. Milbrandt, A. Noelle Larson

**Affiliations:** Department of Orthopedic Surgery, Mayo Clinic, Rochester, MN 55905, USA; cmregan@arizona.edu (C.R.); transtrum.michael@mayo.edu (M.B.T.); bj9r3@mail.umkc.edu (B.J.); milbrandt.todd@mayo.edu (T.A.M.)

**Keywords:** adolescent idiopathic scoliosis, vertebral body tethering, posterior spinal fusion, surgical delay, curve progression, skeletal maturity

## Abstract

**Background**: The implications of delaying surgical intervention for patients with adolescent idiopathic scoliosis (AIS) wishing to undergo vertebral body tethering (VBT) have not yet been explored. It is important to understand how these delays can impact surgical planning and patient outcomes. **Methods**: This was a retrospective review that analyzed all AIS patients treated between 2015 and 2021 at a single tertiary center. Time to surgery from initial surgical consultation and ultimate surgical plan were assessed. Patient characteristics, potential risk factors associated with increased curve progression, and reasons for delay were also analyzed. **Results**: 174 patients were evaluated and 95 were scheduled for VBT. Four patients later required a change to posterior spinal fusion (PSF) due to excessive curve progression. Patients requiring PSF were shown to have significantly longer delays than those who received VBT. Additionally, longer delays, younger age, greater curve progression, and lower skeletal maturity were correlated with significant curve progression (≥5 degrees). **Conclusions**: Surgical delays for AIS patients awaiting VBT may lead to significant curve progression and necessitate more invasive procedures. Patients with longer delays experienced an increased risk of needing PSF instead of VBT. Of those requiring PSF, the majority were due to insurance denials. Optimizing surgical timing and shared decision-making among patients, families, and healthcare providers are essential for achieving the best outcomes.

## 1. Introduction

Adolescent idiopathic scoliosis (AIS) is a three-dimensional deformity of the spine defined as lateral spinal curvature of 10 or more degrees [1,2]. AIS is the most common form of scoliosis affecting 2% to 3% of adolescents ≤16 years of age globally [1,2]. Of these, 0.3% to 0.5% will have a curvature of ≥20 degrees, which is when treatment is generally recommended [1,2,3]. Although the majority of children with AIS will not require any intervention, those with moderate-to-severe curves are at risk of lifelong curve progression and subsequent pulmonary dysfunction, pain, and deformity [1,4]. Curves between 20 and 40 degrees are typically trialed with nonoperative management as a first attempt. In patients with AIS, bracing is the standard of care [5,6]. Patients are instructed to wear the brace for 18 hours per day to prevent further curve progression [5]. However, many curves in this range will continue to increase due to refractory progression or patient noncompliance [5,6]. When nonoperative management fails, surgical intervention is required to correct the deformity, prevent further curve progression, and minimize morbidity [3].

Posterior spinal fusion (PSF) and vertebral body tethering (VBT) are surgical treatment options for AIS. Indications for VBT include skeletally immature patients with AIS and a curve magnitude between 40 and 65 degrees. VBT usually involves the thoracoscopic placement of screws on the convexity of the curve which are then linked by a flexible plastic cord. Through the Heuter–Volkmann principle, there may be an additional correction of the curve as the child grows over time [7,8,9]. Current reports show promising results with several unique benefits differentiating VBT from PSF, including preserved spinal growth, shorter recovery, potentially better functional outcomes, and less motion loss [10,11,12,13,14]. However, VBT is also associated with higher reoperation rates and a lesser curve correction when compared to fusion surgery [12].

The indications for VBT are quite narrow, and not all surgical AIS patients meet inclusion criteria. Patients with large, stiff curves who have inadequate growth remaining are not candidates for VBT. In these cases, PSF remains a reliable and powerful surgical treatment option. Patients with PSF are under less of a time constraint as the procedure can be performed successfully after skeletal maturity and for stiff curves or those greater than 65 degrees [15]. Some patients who are VBT candidates on presentation may have curve progression or complete skeletal growth while awaiting surgery, which results in the loss of the treatment window for VBT. Furthermore, narrowed indications for VBT may reduce reoperation rates, which would suggest that the careful timing of the procedure based on curve magnitude and growth remaining is critical for the long-term viability of this procedure.

Our practice participates in formal shared decision-making strategies to present options of VBT and PSF to patients and parents in a standard, comprehensible fashion [16]. This empowers families to make the best decision based on their preferences and values as well as the current knowledge regarding VBT. However, we noted in our practice that there were significant delays from when a decision for surgery had been made until the surgical date; in some cases, at the preoperative visit, patients were no longer candidates for VBT due to curve progression or advanced skeletal maturity. No prior study has assessed how delays in treatment for patients planning to undergo VBT have affected final surgical intervention and scoliotic progression. We sought to study the impact of surgical delay on our VBT practice and to determine which patients were at the greatest risk of curve progression while awaiting surgery. Furthermore, we aimed to evaluate the rate at which these changes occur in order to better assess a patient’s surgical options.

## 2. Materials and Methods

After IRB approval, we retrospectively reviewed patients with AIS that presented between 2015 and 2021 at a single tertiary referral center and were offered VBT surgery. A medical record data exploration and retrieval tool was utilized to identify patients for inclusion in our study. Patients were included if two sets of radiographs were utilized for decision-making: one used at the consultation visit and another used at the preoperative visit. The consultation visit was defined as the encounter at which surgical intervention was recommended, and the preoperative visit was the encounter immediately prior to surgery. Patients were excluded if the two radiographs were taken less than 28 days apart. Typically, a second radiograph within this interval would be considered unnecessary as the minimal change that might occur within 28 days would be unlikely to alter the surgical plan.

Coronal curvature and Risser sign staging were collected at the two time points. These measurements were agreed upon by two physicians. Bone age using Sander’s simplified skeletal maturity scale (SSMS) was also collected at the time of initial consultation. Finally, any changes to surgical plan and the reasons for surgical delay as mentioned in visit notes were reported.

Primary analysis compared time delay between those who underwent VBT and those with extensive curve progression requiring a change to PSF. Additional analysis involved separating the participants into two groups: those who progressed ≥5 degrees (group A) between the initial consult and surgery and those who did not (group B). Potential risk factors were then analyzed between these two groups, including age, sex, curve type, curve magnitude, Risser, SSMS, and time delay.

Data were evaluated using the GraphPad Prism statistics software version 10.0.2. Standard descriptive statistics, such as the mean and the standard deviation were calculated for patient demographic information. The time delays and risk factors were analyzed for significance using two-tail *t*-tests, Mann–Whitney tests, or Chi-square tests, depending on the type and distribution of the data collected. Two-tail significance levels were reported. Significance was set at α = 0.05.

## 3. Results

During the study period, 174 patients were evaluated for VBT surgery. Furthermore, 95 patients of those evaluated were scheduled for VBT surgery based on surgeon recommendation and patient/family preferences. Ultimately, 91 patients received VBT, and 4 patients received PSF due to curve progression (Table 1).

**Figure 1:**
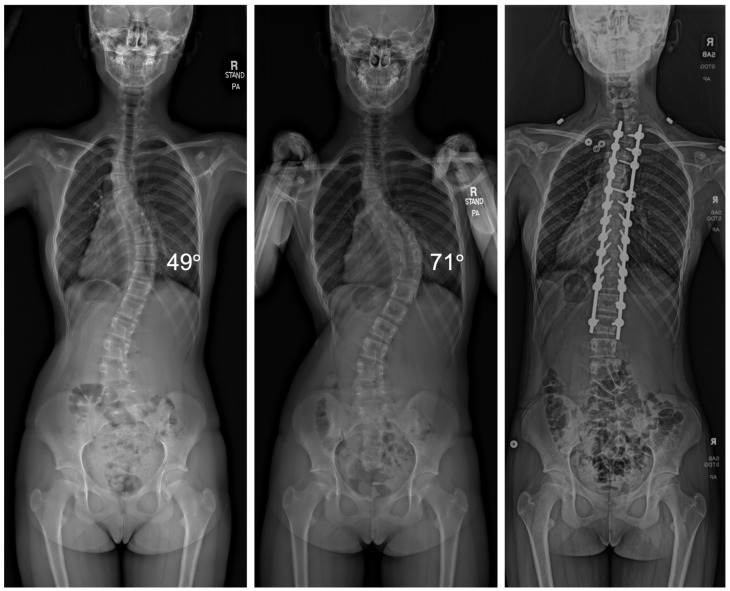
Radiographic imaging of a patient before and after a 6-month surgical delay that resulted in curve progression from 49° to 71°, requiring the transition from VBT to PSF.

Among the 95 patients treated, there was an average curve progression of 5.2 degrees while awaiting surgery. This equated to an average of 1.91 degrees per month. A total of 48 patients of the 95 experienced curve progression of ≥5 degrees. Of the 91 patients that underwent VBT, 66 received thoracic tethers (72.5%), 12 received lumbar tethers (13.2%), and 13 received both thoracic and lumbar tethers (14.3%). Three patients required thoracic PSF, and one patient underwent both thoracic and lumbar PSF (Figure 1).

Patients with VBT with thoracic tethers had an average progression of 4.5 degrees (range: −7 degrees to 32 degrees), while patients with lumbar tethers had an average progression of 2.8 degrees (range: −6 degrees to 12 degrees). Patients with both thoracic and lumbar tethers had an average thoracic progression of 6.2 degrees (range: 2 degrees to 22 degrees) and an average lumbar progression of 5.2 degrees (range: −1 degrees to 21 degrees). Patients that required PSF had an average thoracic progression of 14 degrees (range: 6 degrees to 21 degrees). The single patient with a lumbar curve had a progression of 13 degrees.

### 3.1. Surgical Time Delay

The average time from initial consultation to VBT surgery was 74.8 days (range: 22–246) and 91.9 days (range: 28–245) from initial radiograph to surgical date (Table 2). Initial radiographs were taken an average of 20.8 days (range: 0–100 days) prior to the surgical consultation visit for patients that underwent VBT. Among the four patients that switched to PSF, the average time from initial consultation to PSF surgery was 330 days (range: 164–793) and 361 days (range: 203–792) from initial radiographs to surgery. For patients that underwent PSF, initial radiographs were taken an average of 34.8 days (range: 0–91 days) prior to the surgical consultation visit. Of note, the longest interval of 792 days involved two separate delays: one due to family preference and a second due to COVID-19 concerns. When compared to the VBT group, the patients requiring PSF experienced significantly longer delays in the time from initial consult to surgery (*p* < 0.00001) and from first to second radiographs (*p* < 0.00001).

### 3.2. Risk Factors

When analyzing potential risk factors in patients from group A (curve progression ≥ 5 degrees) compared to group B (curve progression < 5 degrees), we found there was no significant difference in sex, curve type, or Risser scores (Table 3). Curve magnitude at consultation was also found to be insignificant; however, the curve magnitude at the time of surgery and the overall change in curve magnitude were both found to be significantly higher in group A (*p* < 0.00001, respectively). Time from consultation to surgery (*p* = 0.0010) and from first to second radiographs (*p* = 0.0015) were both significantly longer in group A. We also found that group A was made up of younger patients at both the initial consultation (*p* = 0.0094) and surgery (*p* = 0.030). Furthermore, our analysis showed patients in group A had a lower SSMS at the initial visit (0.012).

### 3.3. Reasons for Surgical Delay

The reported delays between consultation and surgery were due to multiple causes, including family preference, availability of surgical dates, insurance, and COVID-19 concerns (Table 4). A total of 59 patients (64%) experienced a delay of 60 days or more between initial consultation and surgery. The most prevalent reason for delay within any time interval was family preference (73%). Family preference included factors such as the time taken by families to consider their surgical options or deferred scheduling due to school, sports, or work.

## 4. Discussion

Adolescent idiopathic scoliosis can progress rapidly if not treated in a timely manner. Patients’ risk of progression is affected by several factors, including the degree and location of the curve, sex, age, and skeletal maturity [17,18]. Prior studies have shown that delays in scheduling fusion surgery can be deleterious for scoliosis patients. It has been shown that 14.8% of patients waiting for more than 180 days had a higher likelihood of additional surgery, such as anterior release, due to curvature progression [19]. Surgical risks and adverse events are also increased when surgical intervention is delayed. Curve progression can result in longer operative times, greater blood loss, and a higher risk of neurological complications [19,20,21,22,23]. Furthermore, delay in surgery can result in longer fusion constructs, decreased surgical satisfaction, and increased postoperative pain [19,24,25,26,27].

Scheduling challenges are common for patients and families planning scoliosis surgery. Arrangements must be made for time off work for parents and caregivers, missed school days for patients, travel to the hospital, and insurance deductibles. For VBT, there is the additional challenge of obtaining insurance pre-approval prior to surgery, which can require multiple appeals as the procedure is not universally covered by insurers. We noticed in our practice that several patients had progression of the curve or increased skeletal maturity, which rendered them ineligible for the planned VBT procedure.

When assessing patient candidacy for VBT, timing becomes increasingly important. In contrast to fusion surgery, the indications for VBT are narrow. Once the patient is past skeletal maturity or the major coronal curvature progresses beyond 65 degrees, VBT is no longer a treatment option according to the U.S. FDA HDE criteria. Most patients that fall outside of these guidelines require PSF to achieve the necessary correction. Four patients in our cohort were initially considered for VBT surgery but were subsequently rescheduled for PSF. One of these patients progressed past the recommended major coronal curvature while waiting for insurance approval and ended up requiring PSF.

There is a long, documented history of the insurance approval process causing delays in treatment [28,29,30]. While some conditions may not worsen during this delay time, others, like scoliosis, face an impending risk of progression and increased surgical morbidity. Prior to FDA approval of VBT in 2019, PSF was the recommended surgical treatment for patients. While delaying PSF by months to years may lead to the necessity for preoperative traction, more extensive procedures with longer surgery times, an increase in the number of vertebrae involved in the construct, and a greater risk for neurological injury during surgery, the operative strategy remains similar regardless of a major coronal curvature at the time of intervention [20,21,22,23]. In contrast, curve progression over just weeks to months can result in a need to change a planned VBT to PSF surgery.

Socioeconomic status and race can also impact access to care in patients with AIS. Prior studies have shown that black patients with public insurance have a higher chance of presenting with initial curves greater than 40 degrees resulting in disparate access to nonoperative treatment [31,32]. Another study showed that curve severity was positively correlated with BMI and negatively correlated with income [33]. While the delays in our patients requiring a change from VBT to PSF were attributed to insurance denials, family preferences, and the COVID-19 pandemic, further research assessing the impact of socioeconomic status on treatment delays is needed.

For patients and families who prefer VBT, a change in surgical plan to fusion surgery can be disappointing. In our four patients, one of the surgical delays was attributed to difficulties in obtaining insurance approval for the procedure. Insurance delays for patients with VBT are a phenomenon not unique to our institution. A plaintiff from Massachusetts recently brought a lawsuit against Anthem Blue Cross and Blue Shield and others for rejecting and ignoring the plaintiff’s request for VBT surgery despite evidence supporting its medical necessity and the plaintiff’s entitlement to coverage [34]. The case was eventually settled out of court.

The study’s retrospective design, relatively small sample size, and data collection at a single tertiary center pose limitations that may impact data accuracy, generalizability, and statistical power. Furthermore, the study lacks the consideration of race and socioeconomic factors and preferences that could also influence measured outcomes. Future research should prioritize prospective, multicenter investigations with larger and more diverse cohorts to mitigate biases, enhance applicability, and strengthen statistical validity. Additional research should be directed toward elucidating common causes of insurance delays and delays due to family preference and can help us to work better with our patients to ensure prompt treatment.

## 5. Conclusions

This study evaluates the impact of surgical delays for patients planning to undergo VBT surgery. It also highlights the challenges of aligning surgical schedules with the optimal treatment window and the potential consequences of missing this window, including transitioning from planned VBT to PSF. In our study, we identified four patients who required fusion instead of VBT due to prolonged delays and extensive curve progression; of these patients, one patient was delayed due to insurance denials, two were delayed because of family preference, and one was delayed due to the COVID 19 pandemic. Given these findings, once the decision has been made to pursue VBT, families should schedule surgery in a timely manner as delaying for an optimum time can put the child at risk for needing fusion surgery, particularly in younger patients. We hope these data may be used to support requests for the expedited review of VBT insurance appeals, as delays in surgical treatment and subsequent changes to planned AIS treatments may adversely affect patient outcomes. Limitations to our study include a small sample size and data collected from a single large tertiary center. Further research into this topic should be conducted on a larger scale.

## Figures and Tables

**Table 1:** Demographics.

	VBT (N = 91)				PSF (N = 4)		
Level	Thoracic	Lumbar	Both		Thoracic	Both	
N	66	12	13		3	1	
Sex (M/F)	12/54	3/9	3/10		1/2	0/1	
Age at Consult	12.8 (1.5)	14 (1.6)	12.8 (1.4)		12.6 (1.5)	12	
Curve at Consult (degrees)	48.6 (6.8)	47.6 (5.2)	T: 45.7 (9.4)	L: 44.7 (8.4)	48.7 (3.5)	T: 40	L: 52
Curve at Surgery (degrees)	50.4 (6.3)	53.1 (8.5)	T: 51.8 (7.2)	L: 49.8 (7)	63 (6.2)	T: 57	L: 65
Change in Curve (degrees)	4.5 (6)	2.8 (4.8)	T: 6.2 (6.4)	L: 5.2 (6.8)	14.3 (7.6)	17	13
Risser at Consult	0.7 (1.1)	1 (1.4)	0.2 (0.6)		1 (1)	0	
SSMS at Consult	3.9 (1.1)	4 (0.9)	3.8 (0.9)		4 (1)	3	

Data presented as mean (standard deviation). T, thoracic; L; lumbar.

**Table 2:** Time to surgical intervention.

	VBT (N = 91)	PSF (N = 4)	*p*-Value
Time from Consult to Surgery	74.8 days (38.6)	329.5 days (309.3)	**<0.00001**
Time from First to Second Radiograph	91.9 days (40.5)	361 days (288.7)	**<0.00001**

Data presented as mean (standard deviation).

**Table 3:** Risk factors for curve progression.

	A: Curve Progression ≥ 5 Degrees (N = 48)	B: Curve Progression < 5 Degrees (N = 47)	*p*-Value
Time from Surgical Consult to Surgery Date (days)	106.2 (111.5)	64.3 (30.8)	**0.0010**
Time from First to Second Radiograph (days)	124.5 (110.6)	81.5 (35.1)	**0.0015**
Age at Consult	12.6 (1.6)	13.4 (1.4)	**0.0094**
Age at Surgery	12.8 (1.7)	13.5 (1.4)	**0.030**
Gender (% male)	22.9%	17.0%	0.52
Risser at Consult	0.6 (1.1)	0.7 (1.1)	0.22
Risser at Surgery	1.1 (1.4)	1.0 (1.3)	0.68
Change in Risser	0.5 (0.8)	0.3 (0.5)	0.17
SSMS at Consult	3.7 (1)	4.1 (1)	0.012
Curve Magnitude at Consult (degrees)	48 (6.6)	48.8 (6.6)	0.70
Curve Magnitude at Surgery (degrees)	57.1 (7.6)	49.5 (6.7)	**<0.00001**
Change in Curve Magnitude (degrees)	9.5 (5.8)	0.7 (2.7)	**<0.00001**
Curve Type (% Thoracic/Lumbar/Both)	Thoracic: 75% Lumbar: 8.3% Both: 16.7%	Thoracic: 72.3% Lumbar: 17% Both: 10.6%	0.37

Data presented as mean (standard deviation).

**Table 4:** Reasons for surgical delay.

	Delay 60–120 Days	Delay 120–180 Days	Delay > 180 Days
Insurance	1	4	1
Family Preference	29	8	2
Availability of Surgical Dates	12	-	-
COVID-19 Concerns	1	-	1

Data presented as number of patients.

## Data Availability

The raw data supporting the conclusions of this article will be made available by the authors on request.

## References

[1] Weinstein S.L., Dolan L.A., Cheng J.C., Danielsson A., Morcuende J.A. (2008). Adolescent idiopathic scoliosis. Lancet.

[2] Weinstein S.L. (2019). The natural history of adolescent idiopathic scoliosis. J. Pediatr. Orthop..

[3] Lenke L.G., Dobbs M.B. (2007). Management of juvenile idiopathic scoliosis. J. Bone Jt. Surg. Am..

[4] Fowles J.V., Drummond D.S., L’Ecuyer S., Roy L., Kassab M.T. (1978). Untreated scoliosis in the adult. Clin. Orthop. Relat. Res..

[5] Karol L.A., Virostek D., Felton K., Jo C., Butler L. (2016). The effect of the Risser stage on bracing outcome in adolescent idiopathic scoliosis. J. Bone Jt. Surg. Am..

[6] Katz D.E., Herring J.A., Browne R.H., Kelly D.M., Birch J.G. (2010). Brace wear control of curve progression in adolescent idiopathic scoliosis. J. Bone Jt. Surg. Am..

[7] Hoernschemeyer D.G., Boeyer M.E., Robertson M.E., Loftis C.M., Worley J.R., Tweedy N.M., Gupta S.U., Duren D.L., Holzhauser C.M., Ramachandran V.M. (2020). Anterior vertebral body tethering for adolescent scoliosis with growth remaining: A retrospective review of 2 to 5-year postoperative results. J. Bone Jt. Surg. Am..

[8] Martin S., Cobetto N., Larson A.N., Aubin C.E. (2023). Biomechanical modeling and assessment of lumbar vertebral body tethering configurations. Spine Deform..

[9] McDonald T.C., Shah S.A., Hargiss J.B., Varghese J., Boeyer M.E., Pompliano M., Neal K., Lonner B.S., Larson A.N., Yaszay B. (2022). When successful, anterior vertebral body tethering (VBT) induces differential segmental growth of vertebrae: An in vivo study of 51 patients and 764 vertebrae. Spine Deform..

[10] Buyuk A.F., Milbrandt T.A., Mathew S.E., Larson A.N. (2021). Measurable thoracic motion remains at 1 year following anterior vertebral body tethering, with sagittal motion greater than coronal motion. J. Bone Jt. Surg. Am..

[11] Crawford C.H., Lenke L.G. (2010). Growth modulation by means of anterior tethering resulting in progressive correction of juvenile idiopathic scoliosis: A case report. J. Bone Jt. Surg. Am..

[12] Hammad A.M., Balsano M., Ahmad A.A. (2023). Vertebral body tethering: An alternative to posterior spinal fusion in idiopathic scoliosis?. Front. Pediatr..

[13] Mathew S.E., Milbrandt T.A., Larson A.N. (2022). Measurable lumbar motion remains 1 year after vertebral body tethering. J. Pediatr. Orthop..

[14] Samdani A.F., Ames R.J., Kimball J.S., Pahys J.M., Grewal H., Pelletier G.J., Betz R.R. (2014). Anterior vertebral body tethering for idiopathic scoliosis: Two-year results. Spine.

[15] de Kleuver M., Lewis S.J., Germscheid N.M., Kamper S.J., Alanay A., Berven S.H., Cheung K.M., Ito M., Lenke L.G., Polly D.W. (2014). Optimal surgical care for adolescent idiopathic scoliosis: An international consensus. Eur. Spine J..

[16] Ifelayo O.I., Brito J.P., Hargraves I.G., Larson A.N. (2021). Development of a shared decision-making tool for adolescents with scoliosis to decide between observation versus fusion surgery. J. Pediatr. Orthop..

[17] Bunnell W.P. (1988). The natural history of idiopathic scoliosis. Clin. Orthop. Relat. Res..

[18] Lonstein J.E., Carlson J.M. (1984). The prediction of curve progression in untreated idiopathic scoliosis during growth. J. Bone Jt. Surg. Am..

[19] Ahn H., Kreder H., Mahomed N., Beaton D., Wright J.G. (2011). Empirically derived maximal acceptable wait time for surgery to treat adolescent idiopathic scoliosis. CMAJ.

[20] Peterson L.E., Nachemson A.L. (1995). Prediction of progression of the curve in girls who have adolescent idiopathic scoliosis of moderate severity: Logistic regression analysis based on data from The Brace Study of the Scoliosis Research Society. J. Bone Jt. Surg. Am..

[21] Suh P.B., MacEwen G.D. (1988). Idiopathic scoliosis in males: A natural history study. Spine.

[22] Weinstein S.L., Ponseti I.V. (1983). Curve progression in idiopathic scoliosis. J. Bone Jt. Surg. Am..

[23] Ylikoski M. (2005). Growth and progression of adolescent idiopathic scoliosis in girls. J. Pediatr. Orthop. B.

[24] Calman R., Smithers T., Rowan R. (2013). Impact of surgical waiting time on paediatric spinal deformity patients. ANZ J. Surg..

[25] Miyanji F., Newton P.O., Samdani A.F., Shah S.A., Varghese R.A., Reilly C.W., Mulpuri K. (2015). Impact of surgical waiting-list times on scoliosis surgery: The surgeon’s perspective. Spine.

[26] Miyanji F., Slobogean G.P., Samdani A.F., Betz R.R., Reilly C.W., Slobogean B.L., Newton P.O. (2012). Is larger scoliosis curve magnitude associated with increased perioperative health-care resource utilization?: A multicenter analysis of 325 adolescent idiopathic scoliosis curves. J. Bone Jt. Surg. Am..

[27] Yang J.H., Bhandarkar A.W., Rathanvelu B., Hwang J.H., Hong J.Y., Modi H.N., Suh S.W. (2014). Does delaying surgery in immature adolescent idiopathic scoliosis patients with progressive curve, lead to addition of fusion levels?. Eur. Spine J..

[28] Gupta A., Khan A.J., Goyal S., Millevoi R., Elsebai N., Jabbour S.K., Yue N.J., Haffty B.G., Parikh R.R. (2019). Insurance approval for proton beam therapy and its impact on delays in treatment. Int. J. Radiat. Oncol. Biol. Phys..

[29] Skaggs D.L., Oda J.E., Lerman L., McGoldrick E., Rice C., Weiss J., Kay R.M. (2007). Insurance status and delay in orthotic treatment in children. J. Pediatr. Orthop..

[30] Yu N.Y., Sio T.T., Mohindra P., Regine W.F., Miller R.C., Mahajan A., Keole S.R. (2019). The insurance approval process for proton beam therapy must change: Prior authorization is crippling access to appropriate health care. Int. J. Radiat. Oncol. Biol. Phys..

[31] Heffernan M.J., Younis M., Song B., Fontenot B., Dewitz R., Brooks J.T., Leonardi C., Barnett S.A. (2022). Disparities in pediatric scoliosis: The impact of race and insurance type on access to nonoperative treatment for adolescent idiopathic scoliosis. J. Pediatr. Orthop..

[32] Zavatsky J.M., Peters A.J., Nahvi F.A., Bharucha N.J., Trobisch P.D., Kean K.E., Richard S., Bucello Y., Valdevit A., Lonner B.S. (2015). Disease severity and treatment in adolescent idiopathic scoliosis: The impact of race and economic status. Spine J..

[33] Laubach L., Sharma V., Alsumait A., Chiang B., Kuester V. (2023). Socioeconomic factors correlation with idiopathic scoliosis curve type and Cobb angle severity. Cureus.

[34] R.M. v Anthem Blue Cross and Blue Shield. 2023, 1:21-CV-12107. https://unicourt.com/case/pc-db5-rm-v-anthem-blue-cross-and-blue-shield-et-al-1102397.

